# Alcohol use disorders and psychiatric diseases in Colombia 

**Published:** 2016-03-30

**Authors:** Hernan G Rincon-Hoyos, Alejandro Castillo, Sergio I Prada

**Affiliations:** 1 Fundacion Valle del Lili Hospital, Cali, Colombia; 2 Escuela de Medicina, Universidad ICESI, Cali, Colombia; 3 Facultad Ciencias Administrativas y Económicas, Departamento de Economía, Universidad ICESI, Cali, Colombia

**Keywords:** Public health, alcoholism, alcohol abuse, alcohol-related disorders, epidemiology, mental disorders, mood disorders, Colombia

## Abstract

**Background:**

An accurate understanding of co-occurrence and comorbidity of alcohol use disorders (AUD) in Colombia is crucial for public health.

**Objective:**

A secondary analysis was conducted, using a 2003/2004 government´s population database to determine the lifetime associations between AUD and other mental and addictive disorders in people of Colombia aged 18-65 years.

**Methods:**

Several statistical analysis were performed: testing prevalence difference in mental disorders by whether the individual had an AUD; a stratified analysis by gender and logistic regression analyses accounting for differences in demographic, socio-economic, behavioral and self-reported health status variables.

**Results:**

People with AUD comprised 9% of the population, of which 88% were males and on average 37 years old. They were more likely to be males, be working, and be current smokers; and less likely to be at home or retired. The population with AUD had greater chance to comply with criteria for all disorders but minor depressive disorder, post-traumatic stress disorder, nicotine dependence, and oppositional defiant disorder.

**Conclusion:**

This study demonstrates a high prevalence of mental disorders in the adult population with AUD in Colombia. The findings highlight the importance of comorbidity as a sign of disease severity and impact on public health and supports the need for training of more professionals and developing appropriate interventions and services.

## Abstract (es)

**Antecedentes:**

Una comprensión precisa de la co-ocurrencia y la comorbilidad de los trastornos por consumo de alcohol (AUD) en Colombia es crucial para la salud pública.

**Objetivo:**

Se realizó un análisis secundario, utilizando una base de datos gubernamental de población 2003/2004 para determinar las asociaciones de vida entre los trastornos por consumo de alcohol y otros trastornos mentales y adictivos en personas de edades comprendidas entre 18 a 65 años de Colombia.

**Métodos:**

Varios análisis estadísticos se realizaron: pruebas de diferencia en la prevalencia de los trastornos mentales en función de si el individuo tenía un AUD; un análisis estratificado por sexo y análisis de regresión logística teniendo en cuenta las diferencias en las variables del estado de salud, demográficas, socioeconómicas, de comportamiento y de auto-reporte.

**Resultados:**

las personas con AUD fueron el 9% de la población, de los cuales 88% eran varones y en promedio tenían 37 años. Estas personas tenían trabajo y eran fumadores activos; y tenían menos probabilidad de estar en casa o ser jubilados. La población con AUD tenía mayores posibilidades de cumplir con los criterios de todos los trastornos, excepto el trastorno depresivo menor, el trastorno de estrés post-traumático, la dependencia a la nicotina, y el trastorno oposicional desafiante.

**Conclusión:**

Este estudio demuestra una alta prevalencia de trastornos mentales en la población adulta con trastornos por consumo de alcohol en Colombia. Los resultados destacan la importancia de la comorbilidad como un signo de gravedad de la enfermedad e impacto en la salud pública y apoyan la necesidad de formación de más profesionales y el desarrollo de intervenciones y servicios apropiados.

## Introduction

Mental and Substance Abuse Disorders have been described worldwide as leading causes in years of life lost to premature mortality (YLLs), to the point that perhaps only through making their prevention and treatment a health priority is possible in a country like Colombia to improve the health of the population 1. 

The World Health Organization (WHO) indicates that approximately 48% of the world population aged 15 years or older consume alcoholic beverages and 16% of drinkers engage in heavy episodic drinking 2. The global burden of harm attributable to alcohol use for different developing world regions is highest for Latin America and the Caribbean, where 9.7% of the Disability Adjusted Life Years (DALYs) and 4.5% of all deaths are attributed to alcohol-related problems 3; while worldwide in 2012 alcohol consumption were estimated to cause 3.3 million deaths (5.9% of all global deaths) and 139 million DALYs 2.

In both developing and developed countries, alcohol use has been shown to cause significant harm to the physical, psychological, and social health of individuals, families, and communities 4,5; and it has also been linked to negative effects on the criminal justice system, employment sector, and economic and social development 6. 

High-income countries have the highest alcohol per capita consumption and the highest prevalence of heavy episodic drinking among drinkers. But, alcohol is used by people of all ages and socioeconomic groups, and drinking starts usually early during adolescence 7,8. People with lower socioeconomic status appear to be more vulnerable to tangible problems and consequences of alcohol consumption 9.

There is substantial evidence to state that excessive alcohol is causally related to risky behaviors and co-morbidly related to the several mental diseases that may differ by gender 10,11, including common violence 12; family violence; traffic accidents; intentional and non-intentional trauma; and sexually-HIV risky behavior; all of them are important public health problems related to alcohol heavy use 13,14. The use of psychoactive substances can occur frequently in conjunction with alcohol, further increasing high-risk behaviors 15. In addition, behavioral disorders, anxiety, depression, mania and other psychiatric disorders are frequently associated to alcohol heavy use 10,16.

The National Epidemiologic Survey on Alcohol and Related Conditions (NESARC) has provided detailed data on comorbidity of Alcohol Use disorders (AUD) with drug use disorders, confirming the high levels of association described in previous studies 17,18. In these studies drug use disorders and comorbidity were more likely to be observed among youth, men, non-married and those of lower socioeconomic status than the AUD-only group. Clinical complexity, costs and disabilities associated with alcohol use disorders, are enhanced by the comorbidity and co-occurrence of other psychiatric disorders, which have been reported in numerous clinical studies 17. 

Information about the co-occurrence and comorbidity of alcohol disorders in Colombia is limited to few reports showing high prevalence 19,20. The Colombian Government decree 3039 of 2007, adopted the National Public Health Plan, stating mental health as one of the priorities and alcohol and substance use control as one of the goals to achieve 8. The Colombian Mental Health Survey (CMHS), conducted in 2003-4, revealed a high lifetime prevalence of anxiety disorders (19.3%), mood disorders (15.0%) and substance disorders (10.6%). Of particular concern, 94.7% of those who had a substance disorder did not use health services 21,22. The last substance use survey done in Colombia in 2013 concluded there is an increase of substance use with regard to previous studies 23, therefore suggesting the need to understand comorbidity. The Colombian Mental Health Survey of 2015 used different approach than in 2003, excluding the determination of frequency of some disorders like Schizophrenia, PTSD and Alcohol and Substance Disorders 24, hence not allowing the possibility to further investigate comorbidity of AUD with all mental disorders. Base on the stated, it could be argue than an accurate understanding of this observable fact is crucial to prevention and development of improved treatment for people who meet criteria for two or more disorders in different clinical settings of developed and developing countries 25. 

This population-based study was conducted to determine the lifetime associations between AUD and other mental and addictive disorders in men and women of Colombia aged 18-65 years, through a secondary analysis of the CMHS of 2003/2004. 

## Materials and Methods

This secondary analysis used the CMHS database 2003-2004 (Colombian Ministry of Health and Social Protection) to obtain information regarding the co-occurrence of mental disorders and AUD. The data was recently released as public use files 26. The survey was conducted in an urban population of 5,526 homes; 4,544 one-on-one interviews with adults were completed with an 87% response rate based on a national level probabilistic, multi-stage, and stratified sample. Survey participants comprised a non-institutionalized population between the ages of 18-65 years with established residency in five Colombian regions. 

The sample released to the public has 4,426 individuals. A full description of sampling procedures and other methodological details on the survey is described elsewhere 21. The survey used internal subsampling to reduce respondent burden by dividing the interview in two parts. Part 1 included core diagnostic assessment (N= 4,426). Part 2 included information about correlates and disorders of secondary interest. All respondents completed part 1. All part-1 respondents who met criteria for any disorder and a subsample of approximately 25% of others were administered part 2 (N= 2,442). 

The survey used a computerized version of the Composite International Diagnostic Interview (CIDI; 15^th^ registered version) that provides diagnoses in agreement with the DSM-IV and ICD-10. Disorder indicators were not released with public use files. To identify those with lifetime prevalence, the authors followed definitions and criteria of the Diagnostic and Statistical Manual of Mental Disorders, Fourth Edition (DSM-IV). 

To test lifetime prevalence difference in mental disorders by whether the individual was alcohol user o not a Fischer´s exact test was conducted. AUD was defined for those with alcohol abuse or dependence according to the original study following international literature. In addition, a stratified analysis by gender was also performed. Lastly, to test whether differences observed in prevalence estimates held after accounting for differences in demographic, socio-economic, behavioral and self-reported health status variables, logistic regression analyses were performed. All analyses were conducted in Stata^®^ 13 using survey data commands. Survey design variables such as primary sample units (PSU) and survey weights were used in all estimations.

Demographic variables included in logistic regressions were age, sex and marital status. The latter was coded as four independent binary variables: married, separated/divorced, widowed and never married. Never married was the reference category. To account for non-linear effects due to age, age is included in levels and to the square.

Socioeconomic variables included in logistic regressions were education and occupation. Education status was coded as elementary, high school dropout, high school, and undergraduate studies (includes both those that finished and those that did not); elementary dropout (including those that never attended) was the reference category. Occupation status was coded as four independent binary variables: working, studying, housewife or retired, and other. Other was the reference category.

Behavioral variables included in regression analyses were obesity and smoking. Obesity/overweight was coded as a dichotomous variable that took the value of 1 for individuals with a BMI (Body Mass Index) greater than 25. Smoking was coded as a dichotomous variable. Self-reported health status variables were "Excellent/Very-Good", "Good" and "Fair or Bad". "Excellent/Very-Good" was the reference category. 

## Results

Table 1 shows socio-demographic and behavioral characteristics on both populations of interest. In particular, people with AUD comprised 9% of the population, of which 88% were males and on the average 37 years old. The predominant marital status was never married (56%) followed by married (31%). Over 30% of these had high levels of education (undergraduate), and 74% reported their occupational status as working. Obesity was a characteristic for almost 8% of this population, and 28% were smokers at the time of interview. Lastly, 41% of people with AUD self-reported their health status as good. 

**Table 1.** Characteristics of survey participants by alcohol use disorders status

	AUD^ ‡ ^	No AUD^ ‡ ^	*p*-value
Demographic characteristics			
Age (Years)	37.0 (1.062)	36.7 (0.299)	0.774
Male (%)	88.2 (0.019)	41.3 (0.012)	<0.001*
Marital status			
Married	30.7 (0.039)	33.5 (0.010)	0.484
Separated/divorced	11.1 (0.023)	9.3 (0.005)	0.389
Widowed	1.9 (0.009)	3.6 (0.004)	0.192
Never married (Reference)	56.3 (0.040)	53.6 (0.011)	0.514
Socioeconomic characteristics			
Education			
Elementary	11.6 (0.021)	13.4 (0.007)	0.446
High School Dropout	24.2 (0.030)	24.1 (0.010)	0.980
High School	16.4 (0.025)	21.7 (0.010)	0.070
Undergraduate	30.6 (0.043)	24.9 (0.012)	0.183
Elementary Dropout (Reference)	17.1 (0.027)	15.8 (0.007)	0.637
Occupation Status			
Working	74.0 (0.038)	54.9 (0.012)	<0.001*
Studying	4.8 (0.030)	7.4 (0.010)	0.497
Home or Retired	9.9 (0.024)	26.9 (0.009)	<0.001*
Unemployed (Reference)	11.3 (0.021)	10.8 (0.008)	0.798
Behavioral characteristics			
Obese	7.7 (0.017)	6.6 (0.005)	0.507
Current Smoker	28.4 (0.040)	13.1 (0.006)	<0.001*
Self-Reported Health			
Excellent/Very Good (Reference)	28.0 (0.033)	34.8 (0.012)	0.065
Good	41.3 (0.039)	41.1 (0.011)	0.960
Fair/Bad	30.7 (0.039)	24.1 (0.010)	0.076
N	325	4,101	

‡Percentage (Standard error)

*Statistically significant (p <0.05)

When compared to the population of alcohol non users, people with AUD were more likely to be males, be working, and be current smokers; and less likely to be at home or retired using a 5% significance level. At a 10% significance level, they also seemed to be less likely to be high school graduates, to be in excellent/good health and more likely to be in fair/bad health.

Table 2 shows prevalence estimates of mental disorders for the total population; Table 3 for males and females, and present statistical tests on differences in prevalence by whether individuals have alcohol abuse/dependency problems. For all individuals and for males, all mental disorders studied were more prevalent for people with AUD with the exception of minor depressive disorders, PTSD, nicotine dependency, and oppositional defiant disorder. For females, five mental disorders were more prevalent among persons with AUD: major depressive disorder, drug abuse, separation anxiety, oppositional defiant disorder, and conduct disorder.

**Table 2.** Lifetime prevalence of mental disorders by alcohol use disorder status

	AUD‡	No AUD‡	p-value
Number of interviewees	325	4,101	
Mood Disorders			
Major Depressive Disorder	17.0 (0.025)	10.2 (0.010)	0.004*
Minor Depressive Disorder	1.7 (0.008)	2.1 (0.003)	0.716
Bipolar I	7.3 (0.031)	1.5 (0.003)	<0.001*
Anxiety Related Disorders			
Agoraphobia	5.2 (0.017)	2.2 (0.003)	0.016*
Posttraumatic Stress Disorder	2.0 (0.008)	1.3 (0.002)	0.346
Substance Use Related Disorders			
Drug Abuse	7.0 (0.016)	0.3 (<0.001)	<0.001*
Drug Dependency	5.2 (0.013)	0.2 (<0.001)	<0.001*
Nicotine Dependency	3.2 (0.014)	1.3 (0.002)	0.053
Other Disorders			
Separation Anxiety	13.6 (0.033)	5.6 (0.004)	<0.001*
Oppositional Defiant Disorder	3.1 (0.014)	1.6 (0.003)	0.137
Conduct Disorder	5.4 (0.013)	1.0 (0.002)	<0.001*

AUD: alcohol use disorder

‡Percentage (Standard error)

*Statistically significant (*p* <0.05). For all variables Fischer's exact test was performed

**Table 3.** Lifetime prevalence of mental disorders for males and females by alcohol use disorder status.

	Males;			Females		
	AUD ‡	No AUD‡	p-value	AUD‡	No AUD‡	p-value
Number of interviewees	265	1,435		60	2,666	
Mood Disorders						
Major Depressive Disorder	14.4 (0.025)	5.7 (0.008)	<0.001*	37.10 (0.076)	13.40 (0.014)	<0.001*
Minor Depressive Disorder	1.9 (0.009)	1.6 (0.003)	0.710			
Bipolar I	7.7 (0.035)	1.2 (0.003)	<0.001*	4.12 (0.023)	1.69 (0.004)	0.143
Anxiety Related Disorders						
Agoraphobia	5.2 (0.019)	0.9 (0.002)	<0.001*	4.73 (0.028)	3.11 (0.004)	0.500
Post-Traumatic Stress Disorder	1.8 (0.008)	0.6 (0.002)	0.074	3.52 (0.028)	1.76 (0.003)	0.402
Substance Use Related Disorders						
Drug Abuse	7.5 (0.177)	0.6 (0.002)	<0.001*	2.82 (0.020)	0.04 (<0.001)	<0.001*
Drug Dependency	5.9 (0.149)	0.2 (0.001)	<0.001*	0.27 (0.002)	0.17 (0.003)	0.689
Nicotine Dependency	3.4 (0.016)	1.6 (0.003)	0.147	2.06 (0.150)	1.13 (0.002)	0.422
Other Disorders						
Separation Anxiety	13.1 (0.037)	3.8 (0.006)	<0.001*	17.53 (0.051)	6.76 (0.005)	0.002*
Oppositional Defiant Disorder	2.8 (0.014)	2.2 (0.005)	0.696	5.86 (0.033)	1.16 (0.002)	0.003*
Conduct Disorder	5.4 (0.013)	1.3 (0.003)	<0.001*	5.75 (0.027)	0.69 (0.001)	<0.001*

‡Percentage (Standard error)

*Statistically significant (p<0.05). For (%) variables Fischer's exact test is reported

Minor Depressive disorder not included because there were no observations for females that have alcohol abuse/dependency problems

Regression analyses are reported in Table 4. The table only reports the OR for the AUD population; nonetheless the regression analyses included all variables described in Table 1 as regressors. In that sense the results reported are ORs after accounting for differences in demographic, socio-economic, behavioral and self-reported health status variables. As shown in Table 4 the population with alcohol AUD were more likely to be diagnosed with all disorders but minor depressive disorder, PTSD, nicotine dependence, and ODD.

**Table 4.** Adjusted Lifetime Odd Ratios of Mental Disorders for Alcohol Use Disorder subjects.

	OR	p-value*	95% CI	
Mood Disorders				
Major Depressive Disorder	2.73	<0.001	1.74	4.29
Minor Depressive Disorder	0.89	0.835	0.31	2.55
Bipolar I	5.14	<0.001	2.33	11.36
Anxiety Related Disorders				
Agoraphobia	3.95	<0.001	1.97	7.91
Posttraumatic Stress Disorder	2.02	0.153	0.77	5.27
Substance Use Related Disorders				
Drug Abuse	12.74	<0.001	5.22	31.08
Drug Dependence	20.72	<0.001	9.60	44.75
Nicotine Dependence	1.92	0.310	0.70	5.27
Other disorders				
Separation Anxiety	3.33	<0.001	1.87	5.90
Oppositional Defiant Disorder	1.48	0.443	0.54	4.01
Conduct Disorder	3.56	<0.001	1.76	7.21

*p-value <0.001. Adjusted by: age, gender, education, occupational status, self-reported health status, obesity and smoker. Smoker was dropped in "Nicotine Dependence" regression for collinearity.

## Discussion

This study, to our knowledge, represents the first analysis of AUD comorbidity with other mental disorders in Colombia. Our results demonstrate a high prevalence of Major Depression, Bipolar I Disorder, Agoraphobia, Drug Abuse, Drug Dependence, Separation Anxiety and Conduct Disorder in the adult population of Colombia with Alcohol Use Disorders, confirming what has been shown elsewhere 27,28. To highlight the importance of these results for Colombia, a recent international report suggested that the "burden of disease attributable to the use of legal substances clearly outweighs the use of illegal drugs and a large part of the substance-attributable burden would be avoidable if known effective interventions were implemented" 29. Furthermore, the problematic use of alcohol is frequently underdiagnosed and undertreated; as a matter of fact, in Latin America, alcohol consumption is 50% greater than the world's mean and almost three-fourths of people who abuse or depend on alcohol have never received any psychiatric treatment 30,31. At this respect, the access to services for mental health disorders in Colombia is low (40%) suggesting a great need of psychiatric care for the severely ill population reported 22,24.

The strong relationship found between bipolar 1 disorder and AUD, has been reported elsewhere to be associated with medication non-compliance, more mixed or dysphoric episodes, and more hospital admissions 32 with great impact to health care cost 17, which further emphasize the necessity for early identification and care in Colombia. As a possible explanation for this relationship, some researchers have hypothesized that alcohol consumption is a form of self-medication 33. The comorbidity found was greater in men than women, but when compared with healthy women, the comorbidity in women was greater than in men 7.

With further analysis by gender, we were able to find a correlation between AUD and major depression in Colombian men and women; and between anxiety disorders and alcohol abuse in men, particularly in those with agoraphobia and social anxiety perhaps reinforcing each other as have been suggested 34,35. At this respect, comorbidity with major mood and anxiety disorders that develop independently of acute intoxication and withdrawal are among the most prevalent psychiatric disorders around the world and have been reported consistently associations between alcohol use disorders and mood and anxiety disorders 3,7,36. 

In the Colombian population, heterotypic comorbidity was found, as well as homotypic comorbidity with other substance dependence disorders, including nicotine dependence 37. Current analyses revealed that homotypic and heterotypic comorbidity were different in men than women, similar to previous reports showing that women drink less alcohol and have fewer problems related to alcohol consumption and AUD 20.

In a cluster analysis of the US National ComorbiditySurvey it was described that three highly co-morbid classes represented 7% of the population and 43.6% of the serious cases were represented by alcohol use disorder co-morbidities 27. Other studies have indicated that alcohol disorder comorbidity with substance disorders with externalizing disorders, could have an underlying etiology contrasting with comorbidity with internalizing disorders like depressive or anxiety disorders that seem to be related to shared etiology factors between the comorbid disorders 38. Disruptive behavior disorders, like conduct disorder or oppositional defiant disorder, have been reported to be the most common co-morbid condition in adolescents with alcohol disorders. Moreover, prospective studies indicate that conduct disorders are the most predictive of subsequent substance use disorder 39.

As an important contribution to understanding and validating the impact of mental disorders comorbidity on physical health in Colombia, in our study the perception of health in the AUD population was found to be lower suggesting greater risk for adverse general health outcomes 40. Comorbidity and co-occurrence of mental disorders and AUD have been associated with greater symptom severity, disability, unrelenting disease course and lower treatment outcome 17,38,41. It should also be noted that this population has been reported to use health services more frequently than the general population 17,38. In addition, comorbidity is frequently found a risk factor for health & HIV risk behaviors 42,43.

The positive health consequences of delaying or moderating the consumption of alcohol use cannot be overstated. It would decrease the incidence of developmental and neurological deficits, traffic accidents, delinquency, psychiatric disorders, high blood pressure, HIV and HIV sex-risk behaviors 44-46. 

Furthermore, lessening alcohol consumption could also improve the likelihood of better physical, emotional development through less interference with how people approach and experience interactions; and also perhaps decrease the incidence of psychiatric and substance abuse disorders, helping to close the reinforcing cycle 47. Appropriately handling of the problem of early drinking should improve the normal delay expected in cognitive and social-emotional development associated with substance use during adolescence resulting in better academic performance, self-esteem, and family and social interactions 48,49. 

Consistent with other studies of AUD and other mental disorders 22,34, the findings suggest that substance disorders are correlated with AUD in Colombians and more prevalent than in the general population. To further investigate is why there were not found associations with posttraumatic stress disorder as previously reported in the literature 48. The findings strongly highlight the importance of comorbidity as a sign of disease severity and supports the need for training of more professionals and developing appropriate interventions and services 50,51.

**Limitations**. The National Survey method used in this study provided quality data in order to run this secondary analysis with the advantage of enabling co-morbidities to be evaluated in a time-efficient manner and without duplication of efforts and at a lower research cost. Nonetheless, it is necessary to mention that this secondary analysis was not an objective of the original survey; therefore, some type of bias can be expected 4. Additionally, the present cross-sectional study design does not allow inferring causality relationships between AUD and other mental disorders. Although the original data is from 2003, the authors do not have a reason to believe that the prevalence of disorders studied could have decreased during these years, since no interventions have been reported, on the contrary according to the substance abuse survey the prevalence of AUD seems to be increasing 23.

## Conclusion

The findings of this study clearly suggest that the co-occurrence of AUD and other mental disorders is prevalent among Colombian men and women, raising concern, and underscoring the importance of providing health services and developing community interventions to confront this public health issue.
